# Vitamin D measurement and effect on outcome in a cohort of patients with heart failure

**DOI:** 10.1530/EC-18-0207

**Published:** 2018-07-24

**Authors:** Federica Saponaro, Alessandro Saba, Sabina Frascarelli, Concetta Prontera, Aldo Clerico, Marco Scalese, Maria Rita Sessa, Filomena Cetani, Simona Borsari, Elena Pardi, Antonella Marvelli, Claudio Marcocci, Claudio Passino, Riccardo Zucchi

**Affiliations:** 1 Department of SurgicalMedical, Molecular and Critical Area Pathology, Laboratory of Biochemistry, University of Pisa, Pisa, Italy; 2 Endocrinology Unit 2University of Pisa, Pisa, Italy; 3 Laboratory of Clinical PathologyUniversity Hospital of Pisa, Pisa, Italy; 4 Fondazione Toscana Gabriele MonasterioPisa, Italy; 5 Institute of Clinical PhysiologyNational Council of Research, Pisa, Italy; 6 Laboratory of EndocrinologyUniversity Hospital of Pisa, Pisa, Italy; 7 Department of Translational Research and of New Surgical and Medical TechnologiesUniversity of Pisa, Pisa, Italy

**Keywords:** vitamin D, 25-hydroxyvitamin D, mass spectrometry coupled to high performances liquid chromatography, immunoassay, hypovitaminosis D

## Abstract

**Objectives:**

The aims of this paper were to evaluate the levels of Vitamin D (VitD) in patients with heart failure (HF), compared to a control group, to assess the effects of VitD on HF outcome and to compare VitD measurement between LIAISON immunoassay and HPLC-MS-MS methods in this population.

**Design and Methods:**

We collected clinical, biochemical and outcome data from 247 patients with HF and in a subgroup of 151 patients, we measured VitD both with LIAISON and HPLC-MS-MS.

**Results:**

HF patients had statistically lower 25OHD levels (45.2 ± 23.7 nmol/L vs 58.2 ± 24.0 nmol/L, *P* < 0.001) and a statistically higher prevalence of VitD insufficiency (61.1% vs 39.5%, *P* < 0.001) and deficiency (24.7% vs 6.6%, *P* < 0.001), compared to healthy controls. There was a significant inverse relationship between baseline 25OHD and risk of HF-related death, with a HR of 0.59 (95% CI 0.37–0.92, *P* = 0.02), confirmed in a multivariate adjusted analysis. Kaplan–Meier survival analyses showed that VitD insufficiency was associated with reduced survival in HF patients (log rank *P* = 0.017). There was a good agreement between LIAISON and HPLC-MS-MS (Cohen’s kappa coefficient 0.70), but the prevalence of VitD insufficiency was significantly higher with the former compared to the latter method (58.3%, *n* = 88 vs 55.6%, *n* = 84, *P* < 0.001). LIAISON underestimated the 25OHD levels and showed a mean relative bias of −0.739% with 95% of limits of agreement (−9.00 to +7.52%), when compared to HPLC-MS-MS.

**Conclusions:**

25OHD levels adequately measured by HPLC-MS-MS showed to be low in HF population and to be correlated with HF-related risk of death.

## Introduction

Heart failure (HF) is a complex and chronic condition and despite advances in therapeutic options, its prognosis remains poor, with high mortality and morbidity. Vitamin D (VitD) has been recently emerged as a factor for HF risk and outcome evaluation (1, 2).

VitD is a fat-soluble vitamin, which functions as a steroid hormone; it is involved in bone and skeletal homeostasis, and it was found to have many others extraskeletal functions, even on cardiovascular system (3, 4, 5, 6). Indeed, hypovitaminosis D is associated with an increased risk of hypertension, coronary artery disease, ischemic heart disease and stroke. Moreover, it is known that it is involved in the pathophysiology of HF, with different mechanisms (downregulation of the renin–angiotensin system, enhancement of insulin secretion and sensitivity, protection against angiogenesis and modulation of inflammatory processes) and low levels of VitD are associated with worse prognosis (7, 8, 9, 10, 11). A limitation in comparing the large amount of literature data on VitD and HF is the variety in VitD status assessment. A near to ideal analytical methods of VitD measurement is strongly required, which could be selective and sensitive, performed using a universal and standardized method and considering the different VitD metabolites (more than 50 VitD physiological products have been described) (5). Traditionally, radioimmunoassay and chromatography have been used for VitD measurement and the majority of routine analyses are performed all over the world by immunoassay techniques, which allow non-chromatographic quantification of total 25 Hydroxyvitamin D (25OHD). In recent years, liquid chromatography-tandem mass spectrometry (LC-MS-MS) has been arisen as the ‘gold standard’ method due to its high sensitivity and accuracy and the possibility to measure multiple VitD compounds at the same time (12, 13).

The aims of this paper are (i) the evaluation of the prevalence of hypovitaminosis by measuring 25OHD levels with HPLC-MS-MS in a cohort of patients with HF compared with a control group of healthy subjects, (ii) the assessment of the effect of VitD on HF outcome, (iii) the comparison of 25OHD measurement between LIAISON immunoassay and HPLC-MS-MS methods in a population of HF patients.

## Materials and methods

### Patients

Between January 2012 and October 2014, we retrieved information about 247 patients with systolic dysfunction (left ventricular ejection fraction, LVEF <50%), referred to the Division of Cardiovascular Medicine of the Fondazione G. Monasterio in Pisa for HF management.

The study was approved by the Local Ethical Committee of Pisa. Consent has been obtained from each patient and healthy subject, after full explanation of the purpose and nature of all procedures used.

All patients fulfilled the European Society of Cardiology (ESC) criteria for the diagnosis of HF and ethical approval was obtained. Each patient underwent comprehensive characterization and the following data were available from basal evaluation: age, gender, BMI, NYHA class, blood pressure, ejection fraction, PTH levels and albumin-adjusted serum calcium.

In all patients, 25OHD levels were measured by high-performance liquid chromatography (HPLC-MS-MS). 25OHD levels were also measure by LIAISON assay in 151 patients, in whom an aliquot of the same sample used for HPLC-MS-MS analyses was available and had been stored at −20°C.

To retrieve data about patient’s follow-up, a telephone survey was conducted: all patients were recalled and data about hospitalizations for HF, major cardiovascular events, worsening of failure and exitus were collected.

### Assays

#### Mass spectrometry coupled to high-performance liquid chromatography (HPLC-MS-MS)

We measured 25OHD levels by HPLC-MS-MS in the whole group (*n* = 245) of HF patients (blood samples – plasma – collected at the baseline evaluation and stored at −20°C). Moreover, we measured 25OHD levels by HPLC-MS-MS in a group of healthy people (*n* = 76) from blood donors of Cisanello Hospital, Pisa.

To validate our HPLC-MS-MS method, we bought from DEQAS (the Vitamin D External Quality Assessment Scheme) a set of samples and compared ‘true’ DEQAS 25OHD values with our measurement. To achieve a good performance, laboratories are required to have 75% of assessable results within ±25% of the true values from DEQAS.

The accurate measurement of 25 hydroxyvitamin D3 (25OHD3) status was carried out by isotope dilution mass spectrometry coupled to high-performance liquid chromatography (HPLC-MS-MS), by using the MSMS VitD Kit from PerkinElmer. It is well known that HPLC-MS-MS offers a good quantification accuracy and the contribution of interfering compounds to the final results is limited (14).

Chemicals: solvents for sample preparation and HPLC elution, such as acetonitrile (LC-MS grade), methanol (LC-MS grade), ultra-pure water (LC-MS grade) and formic acid (for mass spectrometry ≈ 98%), were purchased from Sigma-Aldrich.

Instrumentation: the instrument consisted in an Agilent 1290 Infinity UHPLC system, including autosampler, binary pump and column oven, coupled to an AB-Sciex API 4000 triple quadrupole mass spectrometer (Concord, ON, Canada), equipped with an APCI source. Chromatography was performed by a PerkinElmer Brownlee Supra C18 3 µm, 50 × 2.1 mm HPLC column, protected by a PerkinElmer Brownlee Supra C18 Guard Column.

HPLC-MS-MS conditions: the separation was carried out under gradient conditions by using methanol as solvent A, and water as solvent B, both containing 0.1% formic acid. The MS method, based on positive ion mode multiple reaction monitoring (MRM), made use of the following quantifying transitions: 25OHD_3_, 401.4→159.1; ^2^H_3_-25OHD_3_ (^2^H_3_-25OHD_3_, IS), 404.4→162.1; 401.4→159.1; ^2^H_6_-25OHD_3_ (^2^H_6_-25OHD_3_). Remarkable is the use of ^2^H_6_-25OHD_3_, a surrogate analyte which simulates 25OHD_3_ in the real matrices containing this latter compound, used in the preparation of either calibration standard solutions or quality controls (QCs). Calibration curve and QCs: solutions containing ^2^H_6_-25OHD_3_ at six different concentration levels (4.7, 9.4, 18.8, 37.5, 75, 150 ng/mL) were used as standard solutions to build the calibration curve. They were obtained by reconstituting with water the lyophilized calibrators provided with the kit. Thereafter, a 100 µL aliquot of the resulting solution was added to 200 µL of acetonitrile containing 0.1% formic acid and a suitable amount of IS (daily precipitation solution, DPS). QCs were prepared as described for the calibration solutions, starting from the suitable lyophilized matters provided with the kit. In these solutions ^2^H_6_-25OHD_3_ was at three different concentration levels (9.4, 37.5, 75 ng/mL).

Sample preparation: plasma samples were stored at −20°C. A 100 µL aliquot of thawed sample was added with DPS, as for calibrators and QC.

The intra-assay CV was specified by the producer and was <4.6% while the inter-assay CV was <4.7%

#### Liaison 25OHD total assay

In a subgroup of HF patients (*n* = 151), from whom we had a second aliquot stored at −20°C of the same plasma sample used for HPLC-MS-MS measurement, we quantified 25OHD with LIAISON 25OHD assay, a direct competitive chemiluminescence immunoassay (CLIA), which provides a quantitative determination. VitD binds to its binding protein and during the first phase, (first incubation), it dissociates from it by buffer 10% ethanol and surfactant and binds to the specific antibody on the solid phase. A tracer (VitD linked to an isoluminol derivative) is then added after 10 min. Unbound materials are washed out after incubation. A chemiluminescent reaction starts, when starter reagents are added. A photomultiplier measures light signal as relative lights unit (RLU), which is inversely correlated to the 25OHD concentration.

Assay specificity has been estimated according to CLSI EP 7-A2 guidelines (15). The LIAISON 25OHD assay has an analytical sensitivity of 4 ng/mL. The intra-assay CV was specified by the producer and was 2–4%. The inter-assay CV was 7%. In our laboratory, the imprecision of Liaison 25OHD total assay (DiaSorin) evaluated with 12 control samples throughout 12 months (25OHD concentration from about 19 to 70 ng/mL) ranged from 4.6 to 10.2%.

### Statistical analyses

Categorical variables are expressed as number of cases and percentages. Continuous variables are expressed as mean values and standard deviations or median values and interquartile range (IQR – 25° and 75° quartiles) as appropriate. Difference between groups was tested by chi-square test or paired *t*-test as appropriate. Agreement between LIAISON and HPLC-MS-MS method was measured by concordance correlation coefficient and by graphical display of the data and the reduced major axis of the data (the reduced major axis or SD line goes through the intersection of the means and has slope given by the sign of Pearson’s *r* and the ratio of the standard deviations. The SD line serves as a summary of the center of the data). Bland–Altman plot was used to identify mean bias (the average of the differences between measurements obtained from the two compared assays) and 95% limits of agreement between methods. Cohen’s kappa coefficient (*k*) was used to assess inter-methods agreement for qualitative (categorical) items. To assess the diagnostic performance of 25OHD in cardiac death, the receiver-operating characteristic (ROC) curve analysis was conducted and the area under the curve (AUC), sensitivity, specificity and the best cut off of 25OHD using the highest Youden index were calculated. Cox regression analysis was conducted to examine the hazard ratio (HR) and 95% CI for the association between death and baseline 25OHD. *P* values <0.05 were considered statistically significant. The statistical package Stata (13.0) was used for analysis.

## Results

### HF cohort

The study group included 247 patients, 203 (82.2%) males and 44 (18.8%) females, with a mean age of 65 ± 13 years. All patients had stable HF disease, mostly in the NYHA I and II class (80.2%, *n* = 198) with mean EF of 33 ± 8%. No patients had severe kidney failure (MDRD <15 mL/min); median PTH level was 27.3 ± 18.8 pg/mL (interquartile range 15.8–34.1) and mean albumin-adjusted total serum calcium was 8.9 ± 1 mg/dL. Clinical and biochemical characteristics of patients are summarized in Table 1.

**Table 1:** Clinical and biochemical data of patients in the whole group.

Patients data (*n* = 247)	
Age (years)*	65 ± 13
Male (*n*, %)	203 (82.2%)
Female (*n*, %)	44 (18.8%)
BMI (kg/m²)*	27 ± 5
NYHA	
Class I–II (*n*, %)	200 (80.1%)
Class III–IV (*n*, %)	47 (19.9%)
EF (%)*	33 ± 8
Serum calcium (mg/dL)*	8.9 ± 1
PTH (ng/mL)**	27.3 ± 18.8 (15.8–34.1)

*Values are expresses as mean ± s.d.; **Values are expresses as mean ± s.d. and interquartile range.

In the whole group (*n* = 247) mean 25OHD levels, measured by HPLC-MS-MS were 18.1 ± 9.5 ng/mL (45.2 ± 23.7 nmol/L), VitD insufficiency prevalence according to IOF guidelines (<20 ng/mL or <50 nmol/L) was 61.1% (*n* = 151) and VitD severe deficiency (<10 ng/mL or <25 nmol/L) was 24.7% (*n* = 61).

Patients with 25OHD levels <20 ng/mL (or 50 nmol/L) were more often obese (BMI<30, *P* < 0.01), hypertensive (*P* < 0.05) and in NYHA class 3 (*P* < 0.01) compared to those with 25OHD levels >20 ng/mL. No difference in gender was found between the two groups (*P* = 0.3).

When we compared HF patients with healthy subjects (*n* = 76), the former had 25OHD levels lower than the latter (18.1 ± 9.5 ng/mL or 45.2 ± 23.7 nmol/L vs 23.3 ± 9.6 ng/mL or 58.2 ± 24.0 nmol/L, *P* < 0.001) and a statistically higher prevalence of VitD insufficiency (61.1% vs 39.5%, *P* < 0.001) and deficiency (24.7% vs 6.6%, *P* < 0.001)

During the follow-up period, median 86.5 (63.4–107.3) months, 40 patients (16.2%) died, 26 (10.5%) for HF-related causes. One hundred nine patients (44.1%) were re-hospitalized, 38 (15.4%) underwent major cardiovascular complications and 19 (7.7%) had a sudden worsening of the disease.

In the whole group, there was a significant inverse relationship between baseline 25OHD and risk of HF-related death, with a HR of 0.59 (95% CI 0.37–0.92, *P* = 0.02), but not all-cause mortality (HR 0.85, CI 0.54–1.33, *P* = 0.48). Multivariate Cox regression analysis adjusted for age, gender, BMI and NYHA classes confirmed that 25OHD concentration remained an independent predictor of HF-related death (HR = 0.62, 95% CI 0.39–0.99, *P* = 0.03)

**Figure 1:**
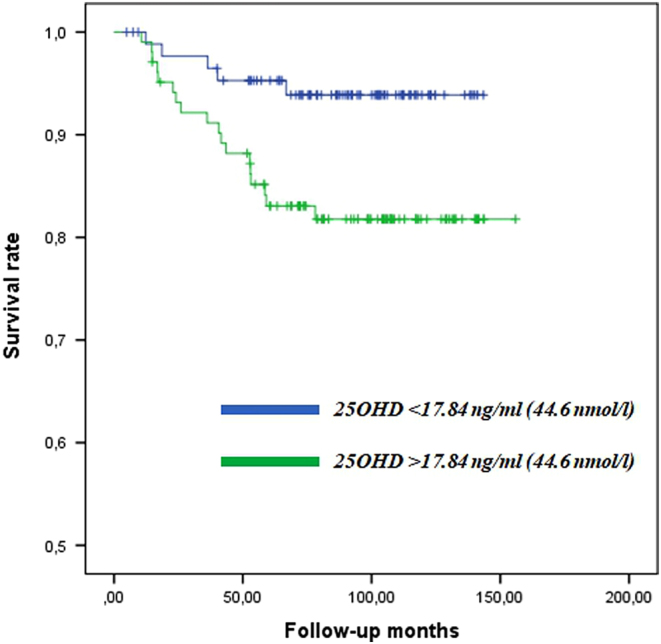
Kaplan–Meier survival analyses according to 25OHD levels <17.8 ng/mL (44.6 nmol/L) showed that VitD insufficiency was associated with reduced survival in HF patients (log rank *P* = 0.017).

At ROC analyses, the AUC values based on 25OHD in HF-related death was 0.66. The best cut-off of 25OHD was set at the highest Youden index and was equal to 17.8 ng/mL (44.6 nmol/L), closed to the clinical threshold, with a sensitivity of 51% and specificity of 79%. Moreover, there was also a significant inverse relationship between baseline 25OHD and the worsening of the failure, after adjustment for age, gender, BMI and NYHA classes (HR = 0.42, 95% CI 0.22–0.81, *P* = 0.010). Kaplan–Meier survival analyses according to 25OHD levels <17.8 ng/mL (44.6 nmol/L) showed that VitD insufficiency was associated with reduced survival in HF patients (log rank *P* = 0.017) (Fig. 1).

### Comparison between the two methods of 25OHD measurement

In 151 patients, of whose blood sample was available, 25OHD levels were assessed either with LIAISON assay or by HPLC-MS-MS, from two aliquots of the same stored plasma sample. No patient was taking any kind of VitD supplementation. HPLC-MS-MS was considered the gold standard and was compared with the true value from DEQAS: 90% of results were within ±25% of the DEQAS-assessed values (Table 2).

**Table 2:** 25OHD measured by DEQAS and by PerkinElmer HPLC-MS/MS from our laboratory.

Sample 25OHD	DEQAS (nmol/L)	PerkinElmer MS-MS Vitamin D Assay (nmol/L)	Variability (%)
1	45.2	35.9	+25.7
2	67.5	79.6	−15.1
3	103.7	109.9	−5.7
4	32.1	32.5	−1.2
5	102.9	119.1	−13.6
6	39.6	39.4	+0.5
7	83.9	93.6	−10.4
8	22.5	23.5	−4.3
9	107.8	128.3	−16
10	55.6	68.7	−19

Serum 25OHD mean values were under the IOF suggested cut-off with both methods; however, when we compared the two methods, 25OHD values measured with LIAISON were statistically lower than values measured with HPLC-MS-MS (18.9 ± 9.4 ng/mL or 47.2 ± 23.5 nmol/L vs 19.6  ± 9.4 ng/mL or 49.0 ± 23.5 nmol/L, *P* = 0.03). When we compared the two methods using clinical thresholds of 25OHD equal to 20 ng/mL (or 50 nmol/L), there was a good agreement between two methods (Cohen’s kappa coefficient 0.70), but the prevalence of VitD insufficiency was significantly higher using LIAISON than using HPLC-MS-MS (58.3%, *n* = 88 vs 55.6%, *n* = 84, *P* < 0.001).

**Figure 2:**
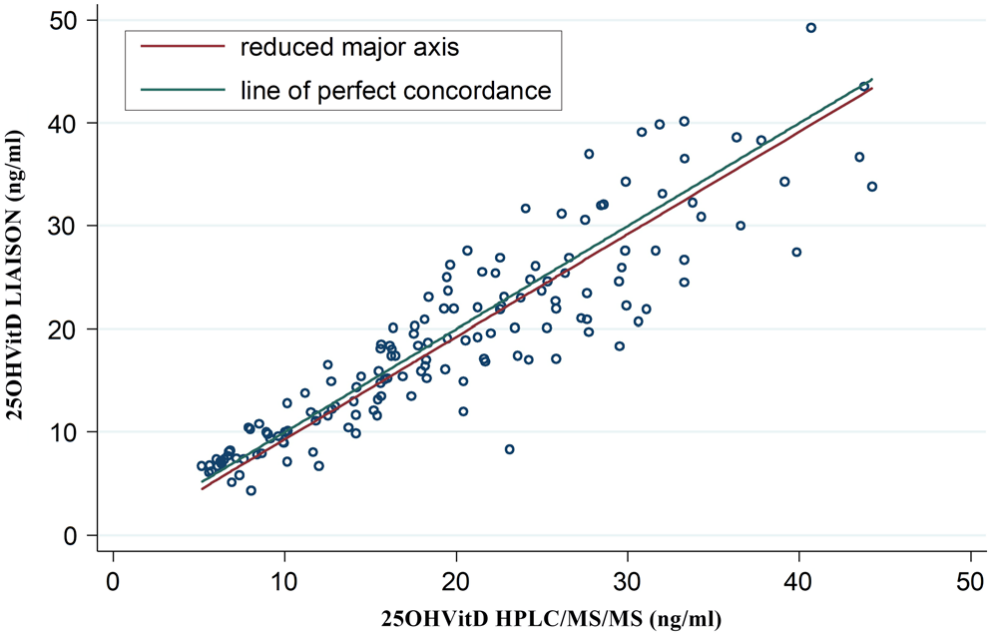
Concordance correlation coefficient of 25OHD measured by LIAISON and HPLC-MS-MS method was 0.896 and the slope between the two methods was 0.995. In the figure (SD line), it is shown that LIAISON method underestimates the 25OHD levels compared to HPLC-MS-MS.

Concordance correlation coefficient of 25OHD measured by LIAISON and HPLC-MS-MS method was 0.896 and the slope between the two methods was 0.995. Figure 2 (SD line) showed that LIAISON method underestimates the 25OHD levels compared to HPLC-MS-MS.

**Figure 3:**
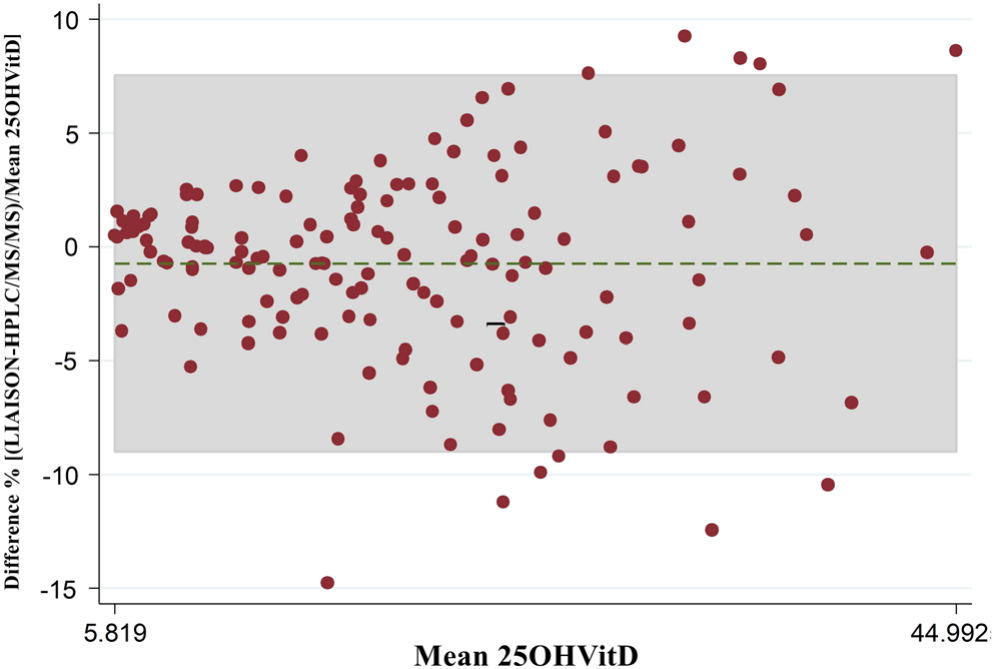
Bland–Altman plots shows percentage difference between 25OHD quantification with LIAISON and HPLC-MS-MS. Green line represents bias of −0.739% (means of paired difference) with 95% of limits of agreement (−9.00 to +7.52%).

Moreover, the inter-assay bias was evaluated by Bland–Altman plot, showed in Fig. 3. LIAISON assay showed a mean relative bias of −0.739% with 95% of limits of agreement (−9.00 to +7.52%), when compared to the HPLC-MS-MS method.

## Discussion

In this study, we demonstrated the high prevalence of hypovitaminosis D in a large cohort of patients with mild-to-moderate HF and the association between 25OHD levels and HF prognosis. Moreover, we showed that 25OHD levels are significantly lower in these populations, compared with matched control group. In our study, 25OHD levels were measured with HPLC-MS-MS, which was demonstrated to be the gold standard for VitD status assessment.

Convincing literature data have been collected about VitD role in pathogenesis and clinical course of cardiovascular diseases, particularly HF. A recent paper from D’Amore *et al*. summarized evidence about the possible mechanisms of VitD effects in HF and clinical studies (16). VitD could exert an antiproliferative role on cardiomyocytes, probably due to the suppression of natriuretic peptide, and it was shown to modulate extracellular matrix turnover in myocardial tissue (17, 18). Moreover, VitD acts as negative regulator of renin-angiotensin-aldosterone system, as was demonstrated in knockout mice (19) and in clinical studies (20). Finally, VitD has a direct non-genomic effect on calcium channel control in cardiomyocytes (21).

Subsequently, clinical cross-sectional and case–control studies have reported association between VitD deficiency and HF: the first study was from Shane *et al*. in 101 patients with severe HF in whom a high prevalence of severe VitD deficiency was demonstrated (26% of patients with 25OHD <37.5 nmol/L or 15 ng/mL) (22). In 2003, Zitterman demonstrated 25OHD levels significantly lower in a population of 54 patients with severe HF, compared to a control group (23). Our study confirms data from previous study, with the novelty that we evaluated a more common HF population with mild-to-moderate HF (prevalent NYHA class I and II), in a larger population and with HPLC-MS-MS 25OHD measurement. Even in the study of Liu *et al*. in which a moderate HF population was described, the prevalence of patients in NYHA class III was higher compared with our study (52% vs 20%), with mean 25OHD levels of 14.6 ng/mL or 36.6 nmol/L (20). Finally, a very large study was conducted by Gotsman *et al*. on 3009 patients with HF, compared with a control group in 2012, but 25OHD levels were measured with immunoassay (10). Our study confirmed a prevalence of severe VitD deficiency (<10 ng/mL or 25 nmol/L) of 24%, similar to that of Gotsman study (28%) and confirmed Gotsman evidence that 25OHD levels are lower in HF patients compared to healthy people (36.9 nmol/L IR 23.2–55.9 vs 40.7 nmol/L IR 26.7–56.9). Other data in literature corroborate this association between 25OHD and HF: in a recent community-based cohort study from Busselton Health Survey, 3946 subjects were followed up for 20 years: there was a significant inverse relationship between 25OHD levels and the risk to develop HF with HR of 0.82 (95% CI, 07–0.95), after adjustments for confounding variables (24).

Some previous studies demonstrated that low 25OHD levels were predictor of poor prognosis and survival in general population (25, 26). However, we showed that this is true in our HF patients' cohort. We confirmed data of Liu and Gotsman that 25OHD is a significant and independent predictor of reduced survival in HF patients (10, 20), but we showed that 25OHD levels are specifically associated with mortality due to HF causes and not with general mortality, after adjustment for confounding variables. In our previous study, we demonstrated a significant inverse correlation between 25OHD levels and MECKI score, even when adjusted in a multivariate model (11) MECKI score is a score of mortality risk validated in HF patients, and it is obtained from six variables independently related to prognosis. These variables include peak VO2% at cardiopulmonary test (CPET), that is reduced in HF patients with poor prognosis and that was correlated with 25OHD levels (9, 11, 27). A possible explanation of the influence of VitD on the outcome of HF patients could be its influence on cardiopulmonary function, and on peripheral skeletal muscle efficiency, with subsequent poor performance at CPET.

In this paper, we also investigated a methodological issue, with the aim to confirm that HPLC-MS-MS should be considered the gold standard for 25OHD measurement.

Indeed, the rising knowledge about VitD's paracrine and endocrine role, beyond calcium homeostasis, has highlighted the need for universal and standardized analytical method for VitD measurement.

Many analytical methods are available for VitD quantification, including competitive protein-binding assays, enzyme-linked immunoassays (ELISAs), radioimmunoassay (RIAs), chemiluminescence assays, high-performance liquid chromatography with UV detection (HPLC-UV), gas chromatography mass spectrometry and more recently liquid chromatography mass spectrometry (LC-MS-MS) (28). LIAISON assay is one of the most widely used and it is a fully automated chemiluminescence assay, which utilize an antibody with the same affinity for 25OHD2 and 25OHD3 and no apparent cross-reactivity for 3epi25OHD metabolite. Moreover, the current version, DiaSorin LIAISON Total, used in this study, was verified to have improved sensitivity and precision (29, 30) and it is approved from FDA.

On the other hand, since 1970, when high-performance liquid chromatography techniques have been introduced, they have been continuously improved and today the most recent LC-MS-MS assay is frequently referred as the ‘gold standard’ method (31, 32, 33). This is due to the high sensitivity, reproducibility and accuracy, and this latter also being influenced by the capability to discriminate 25OHD2 and 25OHD3, as well as their epimeric forms. Moreover, it offers the possibility to measure different VitD metabolites at the same moment (30). In 2009, the use of LC-MSMS was advised by the Nutritional Health and Nutrition Examination Survey (34) in USA and from the UK Food Standard Agency (FSA) in their National Diet and Nutrition Survey (35).

In our study, LIAISON method and HPLC-MS-MS showed a strong correlation, as already reported in other studies (12, 28). LIAISON showed a negative bias (−0.74%) compared to HPLC-MS-MS, even if it was a uniform bias across the range of doses tested. This trend was already described; however, the negative bias in our series was smaller than that in the previous studies. Indeed, in Moon *et al*. study, ADVIA Centaur and LIAISON methods were tested against LC-MS-MS and showed a negative bias of respectively −4.3% and −13.5% (28). To further confirm the reliability of LC-MS-MS, others studies already tested LIAISON and LC-MS-MS against those that were proposed to be the ‘standard’ protocols for VitD measurement. Moon *et al*. used standard reference material (SRM) 972 provided from United States National Institute of Standards and Technology and showed that LC-MS-MS gave the NIST closest results (compared to LIAISON and other methods) (28). Moreover, also The Vitamin D External Quality Assessment Scheme in 2011 confirmed a negative bias for LIAISON against LC-MS-MS of about −4.5% (29).

The negative bias in the correlation analyses, together with the significantly relevant difference in prevalence of VitD deficiency with the two different methods, arises some considerations about the correct clinical evaluation of hypovitaminosis D.

Indeed, in our series, VitD insufficiency (25OHD <50 nmol/L or 20 ng/mL) and deficiency (25OHD <25 nmol/L or 10 ng/mL) prevalence was significantly lower using HPLC-MS-MS, compared to that using LIAISON: that means that probably some patients would be considered as deficient, if tested with chemiluminescence assay and treated with VitD high-dose supplements. The same concern arose in a recent multicenter study in which VitD samples coming from 11 population series were reanalyzed at the University College Cork using the same protocol of LC-MS-MS, certified by CDC’ Vitamin D Standardization Certification Program. They found a discrepancy in the prevalence of hypovitaminosis D in some of the reanalyzed samples: in particular, in the German population, the percentage of patients with 25OHD levels <30 nmol/L was 25.9% with the original method (chemiluminescence) and 15.2% after LC-MS-MS reanalyses (36). Therefore, data from our and other studies, light up the importance of standardization in VitD measurement, for the clinical management and therapy. The most important standardization programs in the world use HPLC-MS-MS, trusting the modern HPLC-MS-MS technology. An additional strength of this study was that we directly tested our HPLC-MS-MS results (the PerkinElmer MS-MS VitD Assay) with DEQAS supplied materials.

Our study has some limitations: we have not the survival follow-up of the control group to compare the effect of VitD on mortality between HF and control group.

In conclusion, this large study on patients with mild-to-moderate HF showed that 25OHD levels are low in this population and significant lower than in healthy people. Moreover, 25OHD is a significant and independent predictor of HF-related mortality.

## Declaration of interest

The authors declare that there is no conflict of interest that could be perceived as prejudicing the impartiality of the research reported.

## Funding

This research did not receive any specific grant from any funding agency in the public, commercial or not-for-profit sector.

## Author contribution statement

Federica Saponaro is directly responsible for experiments, data collection, manuscript draft; Alessandro Saba, Claudio Passino, Claudio Marcocci and Riccardo Zucchi are responsible for supervision of experiments and draft writing, Frascarelli Sabina, Prontera Concetta, Clerico Aldo are responsible for 25OHD measurements, Maria Rita Sessa, Antonella Marvulli, Filomena Cetani, Elena Pardi, Simona Borsari are responsible for healthy controls samples collection, Marco Scalese is responsible for statistical analysis.
